# Evaluation of the Effect of a Live Interview in Journalism Students on Salivary Stress Biomarkers and Conventional Stress Scales

**DOI:** 10.3390/ijerph19041920

**Published:** 2022-02-09

**Authors:** Delfina Roca, Damián Escribano, Lorena Franco-Martínez, Maria D. Contreras-Aguilar, Luis J. Bernal, Jose J. Ceron, Pedro A. Rojo-Villada, Silvia Martínez-Subiela, Asta Tvarijonaviciute

**Affiliations:** 1 Department of Information and Documentation, Faculty of Communication and Documentation, University of Murcia, 30003 Murcia, Spain; delfina@um.es (D.R.); parojo@um.es (P.A.R.-V.); 2 Interdisciplinary Laboratory of Clinical Analysis, Interlab-UMU, Regional Campus of International Excellence ‘Campus Mare Nostrum’, University of Murcia, 30003 Murcia, Spain; det20165@um.es (D.E.); lorena.franco2@um.es (L.F.-M.); marilo.contreras.aguilar@gmail.com (M.D.C.-A.); luisje@um.es (L.J.B.); jjceron@um.es (J.J.C.); asta@um.es (A.T.)

**Keywords:** alpha amylase, cortisol, journalism, saliva, stress, trainings

## Abstract

A career in journalism can be very stressful, as journalists frequently have to deal with uncontrolled situations such as conducting live interviews. Therefore, training is essential during their career, both for the development of communication skills and for the improvement of the real and effective capacity to perform the tasks of their professional activity. The aim of this study was to assess the levels of stress in students before and after a practical training in a professional television set using subjective (State-Trait Anxiety Inventory (STAI) and Likert scale) and objective (salivary cortisol and alpha-amylase) methods. The results indicate that a live interview produces stress in the students as revealed by increased concentrations of cortisol and alpha amylase in saliva. Furthermore, students with lower initial concentrations of these biomarkers obtained better grades in evaluation, suggesting that greater control of anticipatory stress could be associated with a better activity performance.

## 1. Introduction

Journalists usually feel high levels of stress as, in many cases, they must be exposed to the public during the performance of interviews and live shows. This stress can be increased in other situations such as covering news of violence, natural disasters, and accidents [1] or when working under pressure and tight deadlines as companies usually expect journalists to do [2]. Therefore, professional skills acquired during a journalism career, either through practical subjects taken at university or through internships carried out in companies, must effectively shape a graduate profile adjusted to the demand of professionals in this field. This would be the ideal situation, but currently journalism degrees tend to focus on theoretical rather than practical subjects [3]. If practical courses were more common, students’ adaptation to the work environment would be less harmful and would minimise stress and anxiety.

Different authors emphasize that journalism is learned by practising it, ideally in environments similar to those in which the professional practice will be developed [4,5,6]. This is because not all journalism students have innate communication skills for this profession’s performance. The nervousness they suffer before going to a live show in the development of their practice is a very common feeling [7], as is the anxiety of expressing themselves in public [8]. The acquisition of communication skills includes gathering all verbal and non-verbal factors required in human communication, linked with the appropriate way of using them according to the situation in order to develop effective oral and corporal expression through practice [8,9]. A natural expression is also expected from journalists, so that they can evoke emotions in the audience. In addition, security is considered essential to carry out journalism successfully [10]. It contributes to the reduction of stage fright, and also, as Rosa Marín [11] points out, to increased personal confidence, preparation, and mastery of the subject that is going to be discussed.

However, this goal can cause great pressure to the students [12]. Graduates do not usually have the ability to speak in public [13] and the main reason is stage fright. Some of them can feel disproportionate stage fright when they are in front of a camera leading to a state of panic, which is more common in a person who feels subjected to the judgment of others [14]. Indeed, one of the biggest fears individuals have in social situations is public speaking [15]. In such circumstances, expression can become impoverished, making stage fright the most common obstacle in journalistic practice. This is identified as the main obstacle to expressing themselves more freely in the media by different interviewed students [14], even to the point of preventing them from demonstrating their potential communication skills [8].

Therefore, training is essential in a journalism career, both for the development of communication skills and for the improvement of the real and effective capacity to perform the tasks of their professional activity [16]. This is because, as Tabassum and Hossain [17] indicate relating to journalists, a successful future career requires effective oral communication. Improving the oral communication can lead to a more efficient management of stage fright from a theoretical-practical perspective, such as that proposed by Rosa Marín [11], who worked on both a cognitive and behavioural level to encourage students to gain positive experiences and have tools to manage stress. This can even translate into greater job options and greater success.

A convenient way to evaluate and monitor the stress of the students in an objective way can be the measurement of biomarkers of stress in saliva. Saliva is particularly useful because its collection is rapid, easy, non-invasive, and non-stressful. Cortisol and salivary alpha-amylase (sAA) are two of the most studied and used stress biomarkers and have been proven to be useful for monitor stress in university students [18].

The aim of this study was to obtain data on the level of stress in journalism students associated with a practical activity consisting of the performance of live interviews to experts on a professional television set. For this purpose, stress levels before and after such an interview were evaluated using subjective (State-Trait Anxiety Inventory (STAI) and Likert scale) and objective (salivary cortisol and sAA) methods. Furthermore, the relationship between stress levels before the interview and the score obtained for the exercise performed was also evaluated.

## 2. Materials and Methods

### 2.1. Participants

In the present study, students of communication of the University of Murcia, Spain, academic year 2019–2020 were involved. A total of 89 second-year students of the Journalism Degree and fifth-year students of the Joint Program of Journalism and Information and Documentation Degrees (further communication students) attending the compulsory subject ‘Information Production Systems’ were part of the study. The activity was part of an innovation project called ‘Learning to disseminate concepts acquired in the classroom: an interdisciplinary proposal’ and it took place in February and March 2020. Inclusion criteria were attending above noted compulsory subject, signing written consent of participation, completing surveys, and giving a sufficient volume of saliva (>200 µL). The exclusion criteria included rejection to sign written consent of participation, not completing surveys, or giving an insufficient volume of saliva.

### 2.2. Development of the Activity

The activity consisted of conducting a series of interviews spread over three weekdays and performed at a professional television studio: the audio-visual resources building AURED of the University of Murcia. The communication students were divided into groups of 4–5 journalists, a main presenter and 3–4 collaborators (students did not alternate the roles). Each group of students participated only once. All groups were introduced to the activity and its content in a previous practice by the educators (DR, PARV). In addition, one student from the veterinary faculty who participated in a pilot study about promoting communication skills between veterinary and communication students acted as an interviewee [19]. The dynamic of the activity consisted of an introduction by the presenter, who gave way to the collaborators to ask questions of the interviewee. Finally, the presenter made an overall summary of the interview followed by the farewell. The overall activity lasted for 15–20 min. The interventions of all communication students were graded in a 10-grade scale by one of the authors of this study, DR.

Five minutes before (pre-recording) and just after (post-recording) the interview, communication students were asked to complete a survey and give a saliva sample. A survey contained State-Trait Anxiety Inventory (STAI) consisting of 20 questions and the Likert scale (1 to 10), where students had to rate the level of stress they felt at the very moment (1—completely relaxed; 10—very stressed).

### 2.3. Saliva Sample Collection and Analysis

Whole saliva (0.0–1.0 mL) was collected from students by passive drool into a polypropylene tube [20]. The students were instructed not to smoke, drink, eat, or brush their teeth for at least 0.5 h before saliva collection. Tubes with saliva samples were maintained on ice until reaching the laboratory (Interlab-UMU, Murcia, Spain). Once at the laboratory, samples were vortexed and centrifuged (3000× *g*, 10 min, 4 °C). Finally, the supernatant was transferred to 1.5 mL polypropylene tubes and stored at −80 °C until analysis.

Cortisol in saliva was determined using an automated immunoassay system (IMMULITE. Siemens Healthcare Diagnostics. Deerfield, IL, USA) [18]. Intra and inter-assay coefficients of variation (CVs) were below 10% in all cases, and linearity yielded a linear regression coefficient close to 1.

The sAA activity was measured by a commercial kit using the International Federation of Clinical Chemistry and Laboratory Medicine method [18,21]. This was a kinetic spectrophotometric assay that used 4,6-ethylidene(G7)-pnitrophenol (G1)-alpha-D-maltoheptaoside (ethylidene-G7PNP) as a substrate of the enzyme. The intermediate product of substrate hydrolysis reacted with alpha-glucosidase, producing p-Nitrophenol as the final product of the reaction. The rate of p-Nitrophenol formation was directly proportional to the alpha-amylase activity of the sample and could be determined by measuring the absorbance at 405 nm. Reagent volumes were adjusted following the manufacturer’s indications. The assay was adapted to an automatic analyser (Olympus AU2700. Olympus America Inc., Center Valley, PA, USA). The method presented an intra and inter-assay CV below 3% and a linear regression coefficient close to 1.

### 2.4. Statistical Analysis

For the descriptive statistical analysis of the sample, the basic descriptive methods were used, calculating the mean, median, standard deviation (sd), and interquartile range data values using statistical software Microsoft Excel 365 (Microsoft Corp., Redmond, WA, USA) and GraphPad Prism 8 (Software, San Diego, CA, USA).

The normality assumptions were verified with the D’Agostino & Pearson omnibus normality test, given that most data did not follow Gaussian distribution, and non-parametric tests were used, unless otherwise stated. Comparisons for salivary biomarkers, survey data, and grade before and after recording were performed using Wilcoxon matched pairs signed rank test.

Four different groups of students were identified according to sAA and cortisol behaviour in saliva before the activity, and to compare data among the four groups, Kruskal–Wallis test followed by Dunn’s multiple comparison test were performed. Differences among groups were considered statistically significant when *p* < 0.05.

## 3. Results

**Figure 1:**
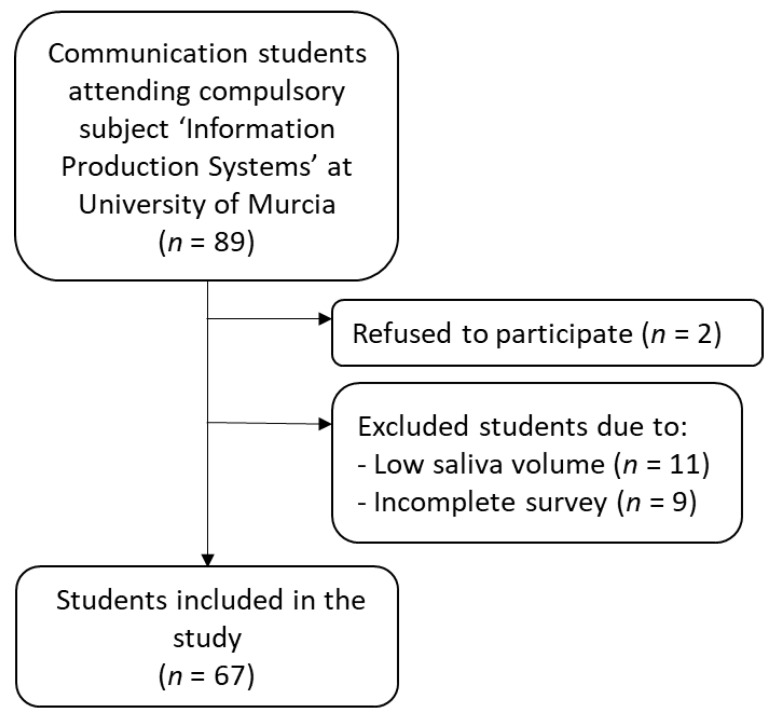
The flow chart of sample inclusion and exclusion.

A total of 67 students (32 (47.8%) women) with ages between 18 and 25 years (mean, 20.4 years) were included in the final study, as they gave written consent for participation, gave sufficient volume of saliva for analyses, and correctly completed questionnaires (Figure 1). The remaining 22 participants were excluded because they refused to participate and sign the written consent (*n* = 2), gave insufficient volume of saliva (<200 µL) at least in one of the samplings (*n* = 11), or did not reply to all questions on the survey (*n* = 9).

**Figure 2:**
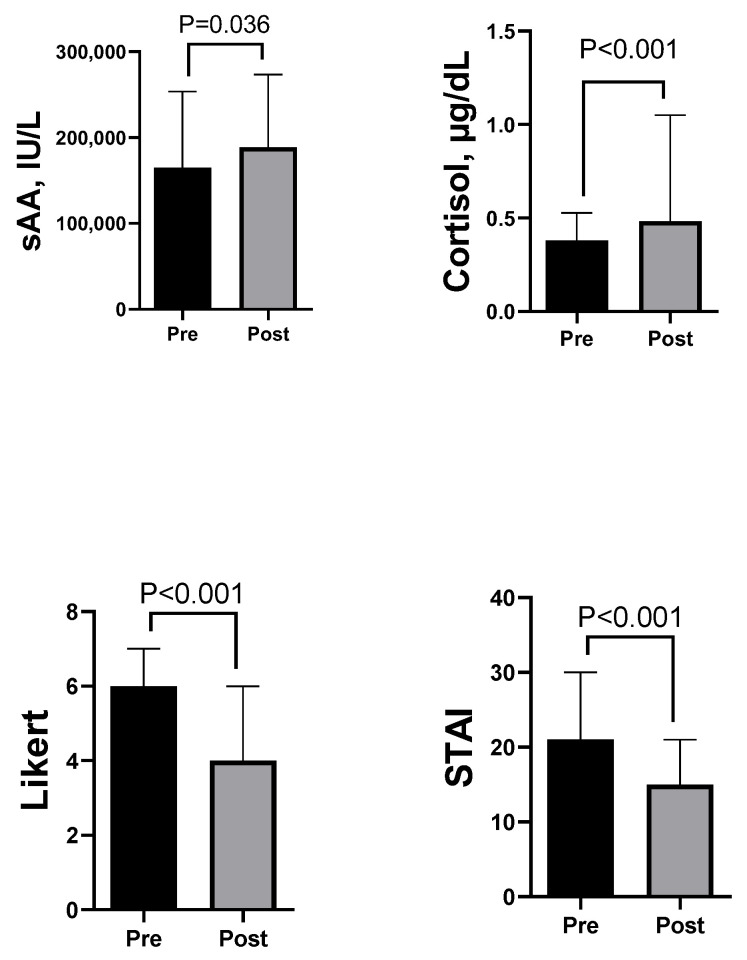
Median and interquartile range data of student salivary alpha amylase (sAA), salivary cortisol, Likert scale, and STAI data of all students (*n* = 67) before (Pre) and after (Post) activity consisting in the live recording of an interview on a television set.

When data from all students were assessed, statistically higher sAA (median (25–75%), (188,600 (104,800–273,330) IU/L) and cortisol (0.48 (0.33–1.05) µg/dL) levels were detected after the practical activity as compared with their levels before it (sAA, 164,860 (106,060–253,510) IU/L; cortisol, 0.38 (0.30–0.53) µg/dL) (*p* < 0.05 and *p* < 0.001, respectively). In contrast, Likert and STAI scores reported by students were statistically significantly lower after the activity (Figure 2).

Four different groups of students according to sAA and cortisol behaviour in saliva before vs. after the activity were identified: Group 1 included students who showed a decrease in both sAA and cortisol after the activity (↓sAA & ↓cortisol); Group 2 included students that showed a decrease in sAA but an increase in cortisol (↓sAA & ↑cortisol); Group 3 included students that showed an increase in sAA and a decrease in cortisol concentrations (↑sAA & ↓cortisol), and Group 4 included students that showed an increase in concentration of both sAA and cortisol after the activity (↑sAA & ↑cortisol).

**Figure 3:**
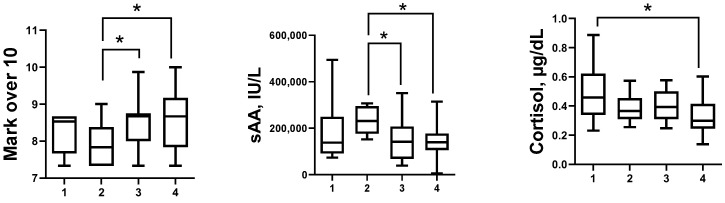
Boxplots representing median, interquartile, and range data of salivary alpha amylase (sAA) and salivary cortisol before activity and the mark received for activity performance of students included in the study. Group 1 included students that showed a decrease in both sAA and cortisol after the activity (↓sAA & ↓cortisol); Group 2 included students that showed a decrease in sAA but an increase in cortisol (↓sAA & ↑cortisol); Group 3 included students that showed an increase in sAA and a decrease in cortisol concentrations (↑sAA & ↓cortisol), and Group 4 included students that showed an increase in concentration of both sAA and cortisol after the activity (↑sAA & ↑cortisol); * *p* < 0.05.

**Figure 4:**
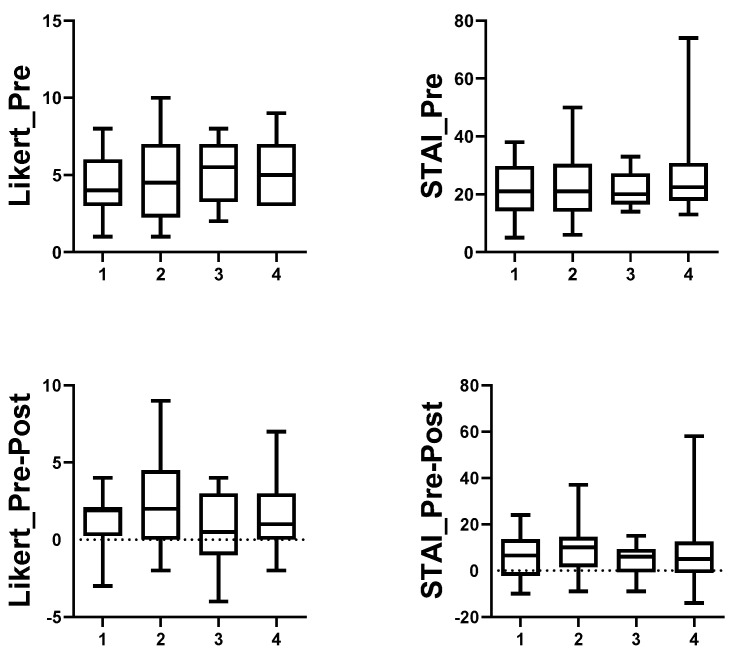
Boxplots representing median, interquartile, and range data of Likert (Likert_Pre) and STAI (STAI_Pre) data before activity; and the difference after-before of Likert (Likert_Pre-Post) and STAI (STAI_Pre-Post) data reported by students. Group 1 included students that showed a decrease in both sAA and cortisol after the activity (↓sAA & ↓cortisol); Group 2 included students that showed a decrease in sAA but an increase in cortisol (↓sAA & ↑cortisol); Group 3 included students that showed an increase in sAA and a decrease in cortisol concentrations (↑sAA & ↓cortisol), and Group 4 included students that showed an increase in concentration of both sAA and cortisol after the activity (↑sAA & ↑cortisol).

When data were evaluated according to sAA and cortisol behaviour in saliva before vs. after the activity, Group 4 (↑sAA & ↑cortisol) showed the lowest median concentrations of sAA (141,470 IU/L; Kruskal–Wallis test, *p* = 0.016)) and cortisol (0.32 µg/dL; Kruskal–Wallis test, *p* = 0.028) before the activity, whereas they obtained the highest marks (median, 8.67 over 10; Kruskal–Wallis test, *p* = 0.040) (Figure 3). No statistically significant differences were detected among groups for data obtained before the activity using the Likert scale or STAI between groups, nor there were differences when changes after-before Likert and STAI data were compared among the four groups (Figure 4).

## 4. Discussion

In this manuscript, the measurements of cortisol and alpha amylase in saliva were used to assess the stress that an academic situation can produce in journalism students. These biomarkers have been used previously to evaluate stress in university students. For example, salivary cortisol and sAA have been measured in psychology students who faced oral examinations and oral presentations, and in veterinary students who faced an oral public presentation made in a non-native language [22,23]. To the authors’ knowledge, this is the first report in which stress is evaluated in journalism students by salivary biomarkers.

In our report, we evaluated the two different components that are involved in the stress response: the hypothalamic-pituitary-adrenocortical (HPA) axis and the sympathetic nervous system (SNS). The HPA axis is a complex neuroendocrine system in which adrenocorticotropic hormone (ACTH) and cortisol are involved [24,25]. The SNS stress response is mediated by catecholamines (epinephrine and norepinephrine) released by cholinergic innervated chromaffin cells in the adrenal medulla [26]. Therefore, both systems are independent and have different physiological mechanisms. Salivary cortisol measurements reflect the free blood circulating cortisol, and it is used to evaluate HPA axis, whereas sAA activity is used to evaluate the SNS because this enzyme is secreted by the parotid gland in response to adrenergic activity [27,28]. In our experimental conditions, when the data of all of students were analysed together, there was a significant increase in cortisol and alpha amylase. This would indicate that the stress associated with the practice produces an activation of both the SNS and HPA axis. Usually cortisol peaks later (20 min after stimulus) than sAA (5 min) in situations of acute stress, so greater increases in cortisol may have been detected if sampling would have been done 20 min after the stimulus.

It is important to point out that there were different responses in cortisol and sAA in the journalism students, with some of them showing increases in both analytes after the practice, others showing changes in only one of the analytes, and even some of them showing decreases in both analytes. The students that showed lower values at the beginning of practice in both sAA and cortisol and an increase after the practice were those that had higher marks for the practice. It could be hypothesized that these students would have less previous stress (because of lower values of the markers) and therefore could better control their initial reactions to the practice with results in a better performance and higher mark. Increases in sAA and cortisol have been previously reported before a stressful practice in university students, possibly because stress was already present due to the students’ knowledge of the task beforehand, and this stress could have led to a reduced performance of the student in the practice and a lower mark [18]. However, this earlier response has not been reported in studies using TSST (Trier Social Stress Test). This difference could be explained by the fact that in this situation, students know about the task beforehand, producing an anticipatory stress response, unlike in TSST-based studies. In the same TSST studies, cortisol response increased earlier when participants were previously informed about the task [29,30].

The lack of relation between cortisol and sAA in saliva and the level of stress indicated by the students in questionnaires was previously reported [18]. This would indicate that objective measurements of stress by salivary biomarkers do not correlate with subjective assessment of stress by questionnaires, and, therefore, the latter should be taken with caution. It is important to point out that, as indicated in the material and method section, the instructors of the course who evaluated the interviews also participated in the research team. This fact may have influenced the level of honesty displayed by students regarding their subjective response to the Academic Stress Inventory, which could have been different if an independent research team completed the study.

This research should be considered as a pilot study, as it had some limitations, such as the relatively low number of participants. Further research with a higher number of participants should be done to confirm our results, and these studies with a larger population would allow for evaluating possible differences in responses between men and women, and for assessing if stress level and the obtained grade depended on time spent practising and overall previous preparation. In addition, ideally a control group of students who did not make the practice should have been included, although this would not allow for the evaluation of the response to stress and the mark obtained.

## 5. Conclusions

Overall, it can be concluded that in our experimental conditions, a practice consisting of a live interview produced an increase in cortisol and alpha amylase in the students that would indicate increased stress. Those students with lower initial values in these biomarkers obtained better evaluations, and this could indicate that the presence of a lower anticipatory stress could be associated with a better performance and obtaining of a higher mark in the evaluation. These biomarkers could be applied to assess other stressful situations that can occur in journalism degree programs and contribute to detect the actions or activities that can produce a higher stress in students and evaluate the possible strategies to reduce it. In addition, these biomarkers could be applied to other University degrees; this can be important in a context in which stress, depression, and anxiety are very common among University students.

## Data Availability

The data presented in this study are available on request from the corresponding author.
